# A Text-Book of Medicine for Students and Practitioners

**Published:** 1887-06

**Authors:** 


A TEXT-BOOK OF MEDICINE FOR STUDENTS AND PRACTITIONERS. By Dr. Adolf Strumpell, formerly Professor and Director of the Medical Policlinic at the University of Leipsic. New York: D. Appleton & Co. 1887.

The fact that Harvard Medical School has already adopted Dr. Strumpell's work as a text-book is enough in itself to put the reviewer in a friendly attitude. A careful examination of the work reveals so much that is good and so little that is bad that a criticism of the book becomes a eulogy.

The author begins with a consideration of the "Acute General Infectious Diseases," the initial chapter being devoted to typhoid fever. He expresses the opinion that the typhoid bacilli discovered by Koch and Eberth are the cause of the disease.

Likewise, in regard to the other infectious diseases, he says that, according to our present information, we must consider them due to a specific, organized, pathogenetic poison. As yet, however, this special cause has not been discovered for each disease.

In the treatment of typhoid fever, he vigorously advocates cold baths. They are to be used often and persistently, though from *three* to *six* a day are enough in many cases. A temperature in the rectum of 103°.6 F. is the indication for resorting to hydrotherapeutics.

This German method of treating typhoid fever, inaugurated and practiced first by Brand and Liebermeister, has never found favor in this country, and probably never will, for the two reasons, first, it entails a great amount of trouble, and second, it has never yielded the brilliant results claimed for it.

If intestinal hemorrhage or cardiac weakness contra-indicates the employment of baths, the internal antipyretics are to be given. He believes that quinine and salicylate of soda will often reduce temperature, but their bad effects on the heart, stomach and nervous system more than counterbalance the good they do. If internal antipyretics must be used, he prefers antipyrine, given in doses of 15 to 30 grains until 90 grains have been taken. He urges the great importance of thorough disinfection of the patient's excreta.

The American editor very wisely inserts a note giving the formulæ of the disinfecting solutions endorsed by the American Public Health Association.

The author gives a few pages to the consideration of idiopathic erysipelas, which he regards as due to a specific virus, residing in the "chain-forming micrococci" of Fehleisen-cholera Asiatica attributed to the infection of the system by a specific micro-organism known as Koch's *comma bacillus;* glanders is attributed to the bacilli discovered by Loffler and Schutz; malignant pustule to the anthrax bacilli, while the part which the *pneumo-coccus* plays in the etiology of lobar pneumonia he regards as still *sub judice.*

The article on tuberculosis is one of the best in the book.

The author takes no uncertain stand in regard to the etiology and pathology of tuberculosis, as the following quotation will show: "The definition of tuberculosis no longer rests upon any external anatomical character. Every disease is tubercular which is excited by the pathogenetic action of a specific kind of bacteria, the *tubercle bacilli* discovered by Koch."

The pathological anatomy of pulmonary tuberculosis is most excellent.

The method of staining the sputa and examining them for the tubercle bacilli is given in detail, so that any one having a proper microscope could mount specimens and recognize the bacilli with ease.

The diseases of the digestive organs are, perhaps, considered rather more briefly than the importance of the subject deserves, though after all this is not a real defect, for each article bristles with valuable information condensed into concise language. The articles on dilatation of the stomach, intestinal obstruction and intestinal parasites are of especial excellence. But the crowning glory of the book is the part devoted to diseases of the nervous system. Three hundred pages, or about one-third of the entire volume, are taken up by the exhaustive and masterly description of these diseases. The labors and writings of such men as Erby Duchenne, Hammond, Wernicke, Charcot, Ecker and Munk have greatly advanced our knowledge in this department, and the author makes free use of their researches, evincing a thorough knowledge of his subject. The text is interspersed with numerous excellent cuts, illustrative of the pathology and clinical features of diseases of the nervous system.

Fifteen pages are devoted to the "Localization of Cerebral Diseases," and here we find all that is known on this subject. By giving all the results of modern research in nervous physiology and pathology, the author makes this part of his book as complete and exhaustive as many special treatises on nervous diseases.

In conclusion it may be said that the book as a whole incorporates all of the valuable additions that have been made to pathology in recent years; it is eminently a text-book for the present day, and cannot be too highly commended to both students and practitioners.

The work of the translators, Drs. Vickery and Knapp, has been conscientiously and intelligently performed, while the editor, Dr. Shattuck, has greatly enhanced the value of the book by many suggestions and explanations of a practical character.

